# Jionoside B1 Sensitizes TNBC to Cisplatin by Inhibiting SIRT3-Mediated Oxidative Stress Defense

**DOI:** 10.3390/biomedicines14020421

**Published:** 2026-02-13

**Authors:** Chenming Xu, Yan Chen, Hao Lin, Yan Chen, Wu Yin

**Affiliations:** 1 State Key Laboratory of Pharmaceutical Biotechnology, College of Life Sciences, Nanjing University, Nanjing 210023, China; dg1830070@smail.nju.edu.cn (C.X.); dg20300070@smail.nju.edu.cn (Y.C.); linhao@jxutcm.edu.cn (H.L.); 2 Jiangsu Cancer Hospital & Jiangsu Institute of Cancer Research, The Affiliated Cancer Hospital of Nanjing Medical University, Nanjing 210009, China; 3 Jiangsu Key Laboratory of Innovative Cancer Diagnosis & Therapeutics, Nanjing 210009, China

**Keywords:** SIRT3, virtual screening, Jionoside B1

## Abstract

**Background:** Sirtuin 3 (SIRT3) is a key mitochondrial regulator that functions as an oncogene in breast cancer, where its overexpression drives chemoresistance. Targeting SIRT3 offers a strategy to overcome resistance mechanisms and improve chemotherapy efficacy. **Methods:** We utilized molecular docking-based virtual screening to identify SIRT3 inhibitors from a natural product library. Candidates were validated via molecular dynamics simulations and binding assays. Efficacy was tested in breast cancer cells and an orthotopic mouse model by assessing cell viability, apoptosis, mitochondrial function, and tumor growth during cisplatin treatment. **Results:** Jionoside B1 was identified as a potent SIRT3 inhibitor that suppresses enzymatic activity, leading to increased SOD2 acetylation. In breast cancer cells, Jionoside B1 significantly enhanced cisplatin sensitivity by promoting ROS accumulation, disrupting mitochondrial potential, and triggering apoptosis. In vivo, the combination of Jionoside B1 and cisplatin inhibited tumor growth more effectively than cisplatin alone. **Conclusions:** Jionoside B1 sensitizes breast cancer cells to cisplatin by inhibiting SIRT3-mediated oxidative stress defense. These findings highlight Jionoside B1 as a promising therapeutic candidate for combination chemotherapy to enhance cisplatin responsiveness in breast cancer.

## 1. Introduction

Triple-negative breast cancer (TNBC) is a highly aggressive subtype of breast cancer characterized by the absence of estrogen receptor (ER), progesterone receptor (PR), and human epidermal growth factor receptor-2 (HER2) expression [1]. Owing to the lack of actionable receptors, systemic treatment relies mainly on conventional chemotherapy, such as cisplatin- or paclitaxel-based regimens. However, despite their widespread use, these therapies benefit only a subset of patients, and their overall efficacy is often limited by the frequent development of chemoresistance and the high metastatic potential of TNBC [2]. Consequently, there remains an urgent need to develop novel strategies that enhance chemosensitivity and improve treatment efficacy in TNBC.

Sirtuin 3 (SIRT3), a NAD^+^-dependent mitochondrial deacetylase, has emerged as a critical regulator of cellular metabolism, oxidative stress response, and energy homeostasis, playing complex and context-dependent roles in cancer biology [3,4,5,6,7]. Unlike its nuclear counterpart SIRT1, SIRT3 exhibits dual functionality in tumorigenesis, acting as either a tumor suppressor or oncogene depending on the specific cancer type and cellular environment [4,8,9,10]. In various cancers, SIRT3 demonstrates oncogenic properties. In non-small cell lung cancer (NSCLC), SIRT3 expression is significantly upregulated in cancerous tissues compared to normal lung tissue, with protein levels positively correlating with malignant biomarkers including Ki-67 and phosphorylated Akt (p-Akt) [11]. Notably, patients with elevated SIRT3 expression demonstrate shorter overall survival, and SIRT3 shows particular oncogenic preference toward squamous cell carcinoma subtypes [11,12,13]. Similarly, in breast cancer, increased SIRT3 transcription levels have been significantly associated with lymph node-positive disease, suggesting its involvement in tumor dissemination and metastatic progression [14,15,16]. Mechanistically, SIRT3’s tumor-promoting effects involve activation of the Akt signaling pathway through post-translational modifications, as evidenced by co-immunoprecipitation and co-localization studies in NSCLC cell lines [17,18,19,20].

Beyond tumor proliferation, SIRT3’s oncogenic role extends to conferring chemoresistance. Through its regulation of metabolic reprogramming, particularly the Warburg effect, and modulation of reactive oxygen species (ROS) levels via deacetylation of MnSOD and other antioxidant enzymes, SIRT3 confers survival advantages to cancer cells under chemotherapeutic stress [9,10,21,22]. Specifically, SIRT3 promotes mitochondrial gene transcription by deacetylating TFAM at K145/K146, enhancing mitochondrial respiration and maintaining ROS homeostasis [23,24,25,26]. In cisplatin (CDDP)-treated triple-negative breast cancer cells, MBD2c recruits SIRT3 to activate TFAM, restoring mitochondrial function and thereby increasing tumor cell resistance to chemotherapy while promoting cell proliferation [25]. Additionally, SIRT3 exhibits protective functions in normal tissues under the same chemotherapeutic insult. In contrast to its pro-survival role in cancer cells, SIRT3 protects kidneys from cisplatin-induced injury by deacetylating mitochondrial DHODH, preventing its degradation and subsequent ferroptosis. Cisplatin exposure causes mitochondrial dysfunction and SIRT3 SUMOylation in renal cells, which reduces SIRT3 deacetylase activity [27]. Interventions such as NMN supplementation or SIRT3 overexpression attenuate DHODH acetylation, lipid peroxidation, and acute kidney injury. This tissue-specific dichotomy highlights the complex and context-dependent nature of SIRT3 function, wherein it promotes cancer cell survival while protecting normal renal tissue from the same cytotoxic agent.

SIRT3 inhibitors have emerged as promising therapeutic agents for specific cancers, particularly those dependent on oxidative phosphorylation (OXPHOS) [12]. Several classes of inhibitors have been developed, including substrate-competitive inhibitors (4′-bromo-resveratrol, YC8-02), nicotinamide-competitive inhibitors (3-TYP, EX-527), and structure-based inhibitors [8,28]. Among these, YC8-02 demonstrates potent SIRT3 inhibition and effectively suppresses lymphomagenesis. By disrupting mitochondrial metabolism, these inhibitors show particular efficacy against hematological malignancies and OXPHOS-addicted tumors [29]. However, their clinical application requires careful evaluation due to SIRT3’s protective roles in normal tissues.

Despite these advances, the discovery of novel SIRT3 inhibitors remains challenging. Virtual screening has become an essential tool to address this need, offering substantial advantages over traditional experimental approaches [30]. Both structure-based and ligand-based methods can accurately predict protein-ligand binding affinities, with state-of-the-art platforms achieving hit rates exceeding 40% in prospective studies [31]. The integration of artificial intelligence and machine learning has further enhanced screening performance, allowing researchers to efficiently explore chemical spaces that would be impractical to test experimentally. Together, these technological advances make virtual screening a cost-effective and scalable strategy for discovering novel SIRT3 inhibitors and other therapeutic candidates against challenging targets. In this study, we identified candidate Jionoside B1 from a natural product library through molecular docking-based virtual screening and further elucidated its binding mode with SIRT3 using molecular docking and molecular dynamics simulations. Microscale thermophoresis (MST) assays confirmed that this compound exhibits favorable binding affinity with SIRT3. Moreover, both in vitro and in vivo experiments demonstrated that Jionoside B1 significantly enhances tumor cell sensitivity to cisplatin. Our findings indicate that inhibition of SIRT3 activity by Jionoside B1 represents a promising therapeutic strategy for cancer treatment.

## 2. Materials and Methods

Reagents and antibodies

Cisplatin and Jionoside B1 were purchased from TargetMol (Shanghai, China). The Annexin V-FITC/PI Apoptosis Detection Kit was purchased from Yeasen Biotech (Shanghai, China). The following antibodies were used in this study: PARP (9532; used for Western blot analysis), cleaved PARP (5625; used for immunohistochemistry assays), Bcl-xL (2764) (CST, Danvers, MA, USA); Acetyl-SOD2 (K68) (DY1546), Cytochrome c (CY5734) (Abways, Shanghai, China); Bax (YM8175), SOD2 (YM8048), SIRT3 (YM8158) (Immunoway, Beijing, China); Bcl2 (120158) (Zenbio, Chengdu, China); Caspase 3 (19677-1-AP) (Proteintech, Wuhan, China). Recombinant Human SIRT3 (Ag26280, Proteintech, Wuhan, China).

Bioinformation analysis

The gene and protein expression levels of SIRT3 in breast cancer patients and healthy controls were retrieved from the UALCAN database [32]. Data for the METABRIC cohort were accessed via cBioPortal, and datasets GSE230881, GSE123845 and GSE25066 were obtained from the GEO database [33,34,35,36]. For the GEO datasets, raw FPKM matrices were converted to TPM and log2-transformed, with expression differences assessed by the Mann–Whitney test. Survival analyses were performed using Kaplan–Meier curves and the log-rank test. Overall survival was defined as the duration from baseline to death or last follow-up. Patients were stratified by SIRT3 expression levels using either the median value or an optimal scanning cut-point method to maximize statistical significance while ensuring adequate group sizes. HRs (95% CIs) were determined using Cox proportional hazards models. All analyses were executed in R (version 4.4.1).

The molecular docking-based virtual screening

The high-resolution X-ray crystal structure of SIRT3 (PDB code: 4JSR) was selected for molecular docking-based virtual screening in this study, owing to its well-defined co-crystallized ligand binding mode. The screening library consisted of 3011 molecules extracted from a traditional Chinese medicine monomer library database. Molecular docking was performed using the AutoDock Vina (version 1.2.5) algorithm with default parameters [37]. Finally, the top-ranked compounds were selected for further evaluation.

Molecular dynamics

Molecular dynamics (MD) simulations were performed using GROMACS (v2021.03) with the OPLS4 force field to capture the dynamic nature of protein–ligand interactions beyond the static binding modes obtained from virtual screening [38]. The protein–ligand complex was placed in a rectangular box solvated with SPC water, with a 10 Å buffer distance, and the system was neutralized by adding appropriate counterions. Energy minimization was carried out to optimize the system configuration and remove unfavorable contacts. Subsequently, a 100 ns simulation was conducted in the NPT ensemble at 300 K and 1.01325 bar. After the simulation, trajectory analyses were performed to evaluate structural stability and characterize protein–ligand interactions.

Microscale thermophoresis

MST experiments were performed using a Monolith NT.115 instrument (NanoTemper Technologies, Watertown, MA, USA) following established protocols. Purified SIRT3 protein was fluorescently labeled with a RED-NHS Labeling Kit (NanoTemper Technologies) at a final concentration of 5 nM. Jionoside B1 was serially diluted across 16 concentration steps in the same buffer as the SIRT3 protein to maintain consistent experimental conditions. MST measurements were carried out at a constant temperature of 25 °C, using a 5 s LED on-time followed by a 30 s MST on-time. Data acquisition and analysis were conducted with the MO. Affinity Analysis software (version 2.3) NanoTemper Technologies).

Cell lines and cell culture

Human breast cancer cell line MDA-MB-231 and mice breast cancer cell line 4T1 were obtained from the Cell Bank of the Chinese Academy of Sciences (Shanghai, China). Cells were maintained in Dulbecco’s Modified Eagle Medium (DMEM) supplemented with 10% fetal bovine serum at 37 °C in a humidified atmosphere containing 5% CO_2_.

CCK-8 Assays

4T1 or MDA-MB-231 cells were seeded in 96-well plates at a density of 5 × 10^3^ cells per well and allowed to adhere overnight. Experimental groups were treated with specific agents. After 24 h of incubation, cell viability was evaluated using the Cell Counting Kit-8 (CCK-8) assay. Specifically, 10 µL of CCK-8 reagent was added to each well and incubated for 1 to 2 h at 37 °C. Absorbance was then measured at 450 nm using a microplate reader to quantify viable cells based on metabolic activity.

Intracellular ROS assay

Intracellular reactive oxygen species (ROS) levels were determined using a ROS Assay Kit (Beyotime, Shanghai, China). MDA-MB-231 cells were seeded in 96-well plates at a density of 5 × 10^3^ cells per well and incubated overnight to allow attachment. Following treatment with the indicated agents for 6 h, cells were stained with DCFH-DA according to the manufacturer’s protocol. Fluorescence intensity was measured using a microplate reader at an excitation wavelength of 488 nm and an emission wavelength of 525 nm.

Mitochondrial membrane potential (MMP) measurement

Mitochondrial membrane potential (MMP) was assessed using a JC-1 Assay Kit (Beyotime, Shanghai, China) according to the manufacturer’s instructions. Briefly, MDA-MB-231 cells were treated with Jionoside B1 (10 µM), cisplatin (20 µM), or their combination for 12 h. Cells were then harvested with 0.25% trypsin and washed twice with PBS. The cells were resuspended in a mixture of 500 μL culture medium and 500 μL JC-1 staining solution, followed by incubation at 37 °C for 20 min in the dark. Subsequently, the cells were washed three times with cold staining buffer and analyzed by flow cytometry.

Flow cytometry measurement of cell cycle

MB-231 cells were treated with Jionoside B1 (10 µM), cisplatin (20 µM), or their combination for 12 h, then harvested and washed with PBS, and fixed in 70% ice-cold ethanol dropwise and stored at −20 °C for 2 h. After fixation, cells were washed twice with PBS and resuspended in PI staining solution (50 µg/mL propidium iodide, 100 µg/mL RNase A in PBS). Samples were incubated for 30 min at room temperature in the dark. DNA content was analyzed on a flow cytometer using linear fluorescence, and cell-cycle distribution was quantified.

Flow cytometry measurement of apoptosis cells

Apoptosis was analyzed using an Annexin V-FITC/PI apoptosis detection kit (Yeasen Biotechnology, Shanghai, China) following the manufacturer’s protocol. Briefly, MDA-MB-231 cells were treated with Jionoside B1 (10 µM), cisplatin (20 µM), or their combination for 24 h, then harvested using 0.25% trypsin for 5 min at 37 °C and washed twice with PBS. Following centrifugation at 300× *g* for 5 min at 4 °C, cells were resuspended in binding buffer containing Annexin V-FITC and propidium iodide and incubated for 15 min at room temperature in the dark. Annexin V+/PI− cells represented early apoptosis, while Annexin V+/PI+ cells indicated late apoptosis or necrosis.

Protein extraction and Western blot analysis

Proteins were extracted from cells using RIPA lysis buffer (Beyotime, Shanghai, China) supplemented with a protease inhibitor cocktail, and protein concentrations were determined with a BCA protein assay kit (Fude Biological Technology, Hangzhou, China). Equal amounts of protein were separated by sodium dodecyl sulfate–polyacrylamide gel electrophoresis (SDS-PAGE) and transferred onto polyvinylidene difluoride (PVDF) membranes (0.45 μm; Millipore, Billerica, MA, USA). The membranes were blocked with 5% nonfat milk and incubated with specific primary antibodies overnight at 4 °C. After washing with PBST, the membranes were incubated with HRP-conjugated secondary antibodies (1:50,000; Bioworld, Nanjing, China) and visualized using an ECL kit (Sparkjade, Jinan, China). Chemiluminescent signals were captured with Tanon imaging software (Version 1.0) (Beijing, China), and band intensities were quantified using ImageJ software (Version 1.48).

Antitumor activity in vivo

Specific pathogen-free (SPF) female BALB/c mice (5 weeks old, 18–20 g) were obtained from Anuokang Biotechnology Co. (Nanjing, China). All animal procedures were approved by the Institutional Animal Care and Use Committee of Nanjing University (Approval No. IACUC-2501008) and conducted in strict accordance with institutional guidelines. To establish the orthotopic breast cancer model, 4T1 (5 × 10^6^) cells were injected into the mammary fat pads of the mice. Seven days post-inoculation, when the average tumor volume reached approximately 100 mm^3^ the mice were randomized into four groups: (1) Vehicle control (PBS, i.p.); (2) Jionoside B1 (5 mg/kg, i.p.); (3) Cisplatin (2 mg/kg, i.p.); and (4) Combination group (Jionoside B1 5 mg/kg + Cisplatin 2 mg/kg, i.p.). Tumor volume was measured using the formula: length × width^2^/2. At the end of the experiment (14 days after the first administration), the animals were sacrificed, and the tumors were collected for further experiments.

Immunohistochemical analysis

Immunohistochemical analysis of the paraffin tissue sections obtained from the tumor tissues of mice was performed following the protocol previously reported [39]. Briefly, sections were deparaffinized, rehydrated, and subjected to antigen retrieval in citrate buffer (pH 6.0). Endogenous peroxidase activity was blocked with 3% hydrogen peroxide, followed by blocking with 5% normal serum. Sections were incubated overnight at 4 °C with the primary antibody cleaved PARP (1:100). After washing, sections were treated with HRP-conjugated secondary antibodies and developed with DAB. Nuclei were counterstained with hematoxylin. Finally, the stained slides were scanned using a whole-slide imaging system (Olympus VS200, Olympus, Tokyo, Japan).

Statistical analysis

Data are presented as mean ± standard error of the mean (SEM). Group comparisons were performed using one-way analysis of variance (ANOVA), with two-group comparisons analyzed using *t*-tests. Statistical significance was defined as *p* < 0.05. All analyses were performed using GraphPad Prism 10 software.

## 3. Results

### 3.1. SIRT3 Is Overexpressed in BRCA and Is Associated with Poor Overall Survival and Chemotherapy Sensitivity

**Figure 1:**
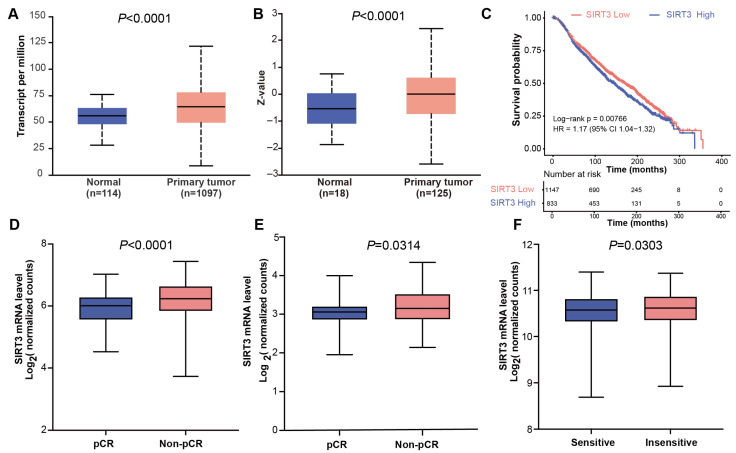
SIRT3 is overexpressed in BRCA and is associated with poor overall survival and chemotherapy sensitivity. (**A**) Expression of SIRT3 in normal and BRCA samples. (**B**) Protein level of SIRT3 in normal and BRCA samples. (**C**) The correlation between SIRT3 expression and overall survival in BRCA samples from METABRIC cohort. (**D**) SIRT3 expression in pCR and non-pCR groups in dataset GSE230881. (**E**) SIRT3 expression in pCR and non-pCR groups in dataset GSE123845. (**F**) SIRT3 expression in chemotherapy-sensitive and chemotherapy-insensitive groups in dataset GSE25066.

Analysis of the UALCAN database showed that SIRT3 mRNA and protein levels are significantly upregulated in breast cancer tissues compared with normal tissues (Figure 1A,B). Consistently, METABRIC data indicated that patients with high SIRT3 expression have significantly worse overall survival than those with low expression (Figure 1C).

To further investigate the clinical relevance of SIRT3 across breast cancer subtypes, we analyzed SIRT3 expression and prognostic value in Luminal (ER+/HER2−), Luminal-HER2 (ER+/HER2+), HER2-enriched (ER−/HER2+), and Triple-Negative Breast Cancer (TNBC) using METABRIC cohort. SIRT3 expression levels showed no significant differences among the four subtypes (Figure S1A), suggesting ubiquitous expression across breast cancer. Importantly, Kaplan–Meier survival analysis revealed that high SIRT3 expression was consistently associated with worse overall survival across all subtypes (HR > 1.0 in all groups), with statistical significance achieved in Luminal (*p* = 0.015, HR = 1.19) and TNBC (*p* = 0.047, HR = 1.32) cohorts (Figure S1B).

Additionally, analysis of three NAC-related GEO cohorts (GSE230881, GSE123845 and GSE25066) revealed that lower SIRT3 expression was associated with chemotherapy sensitivity and pathological complete response (pCR) (Figure 1D–F). Collectively, these findings indicate that SIRT3 is closely associated with breast cancer development and prognosis, suggesting its potential as a promising therapeutic target for cancer treatment. Given the aggressive nature of TNBC and the urgent need for effective chemosensitization strategies in this subtype, we selected TNBC models for subsequent functional validation.

### 3.2. Molecular Docking-Based Virtual Screening for SIRT3 Inhibitor

**Figure 2:**
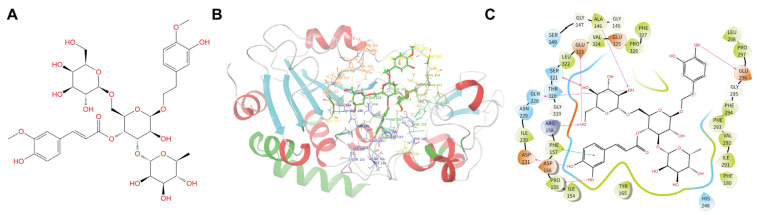
Modes of Jionoside B1 binding to SIRT3 revealed through molecular docking analysis. (**A**) Chemical structure of Jionoside B1. (**B**) Overall binding sites and the interaction pattern of Jionoside B1 in the SIRT3 pocket. (**C**) Two-dimensional interaction between Jionoside B1 and SIRT3.

Molecular docking is a robust and extensively validated approach in virtual screening for identifying candidate compounds [37]. Consequently, this study utilized molecular docking to screen a traditional Chinese medicine monomer library for potential SIRT3 inhibitors. Table 1 summarizes the top 40 candidates ranked by docking score. Notably, the top 20 compounds exhibited scores lower than −11, suggesting a potentially high binding affinity with SIRT3. Among these candidates, Jionoside B1, a phenylpropanoid derived from the herb Eriophyton wallichii, achieved the highest docking score and was therefore selected for subsequent experimental validation (Figure 2A). Binding mode analysis revealed that Jionoside B1 fits well within the SIRT3 active site, forming multiple key intermolecular interactions with residues Ala146, Pro155, Phe157, Arg158, Gln228, Asp231, Glu296, Ser321, and Glu323 (Figure 2B,C). These structural insights provide a solid theoretical basis for the specific inhibition of SIRT3 by Jionoside B1.

### 3.3. Molecular Dynamics Simulation Analysis

**Figure 3:**
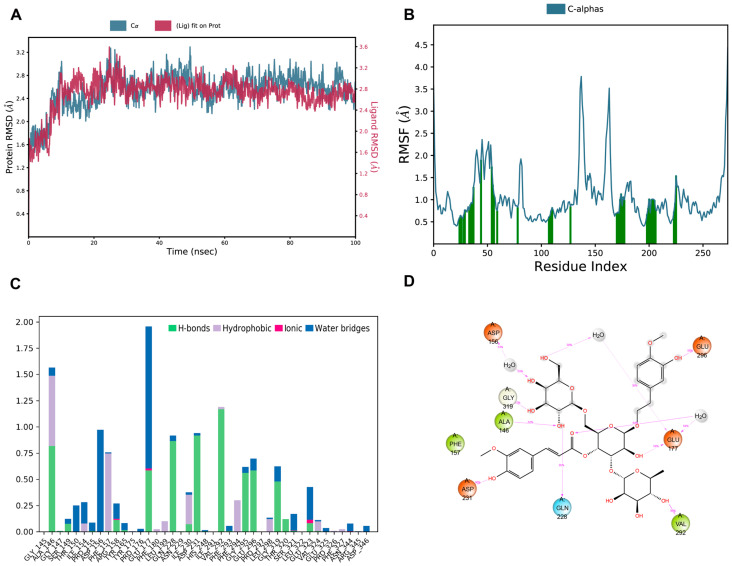

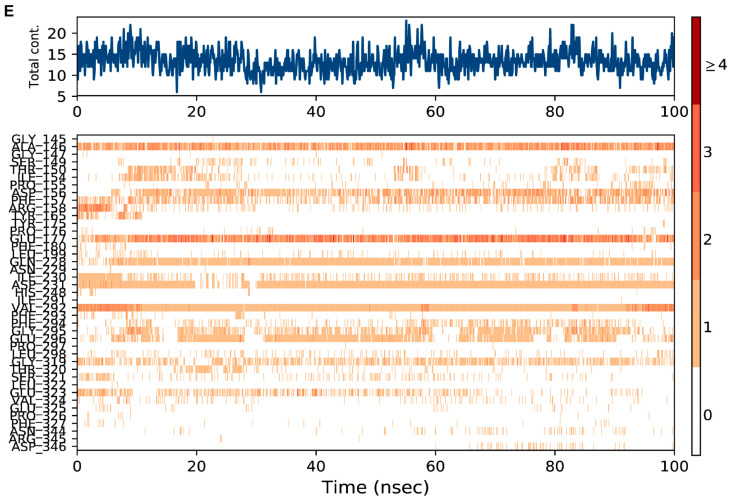
Molecular dynamics simulation analysis of SIRT3-Jionoside B1 complexes. (**A**) Time-dependent RMSD of the MD trajectory. Blue: Protein Cα RMSD. Red: Ligand RMSD with respect to the protein backbone. (**B**) Per-residue RMSF analysis revealed localized flexibility patterns across the protein structure. Green vertical bars indicate residues that interact with the ligand. (**C**) Interaction fractions of the contacts each residue formed with SIRT3-Jionoside B1 over the course of the molecular dynamics trajectory. (**D**) Two-dimensional interaction between Jionoside B1 and SIRT3 over molecular dynamics trajectory. (**E**) Protein–ligand contact analysis for the SIRT3-Jionoside B1 complex over a 100 ns MD simulation. Total number of protein–ligand contacts as a function of time (up). Per-residue contact heatmap. Color scale indicates the number of contacts (down).

A 100 ns molecular dynamics simulation was performed to further analyze the trajectory data, which contributed to understanding the stability and expansion of the ligand–protein complex. Both the protein backbone (Cα) and the ligand RMSD trajectories reached a stable plateau after an initial equilibration phase, indicating that the ligand maintained a stable binding pose within the active site (Figure 3A). RMSF analysis indicates that the majority of residues in the SIRT3 structure remained rigid with fluctuations below 2.0 Å, while higher flexibility was observed primarily in loop regions and at the termini (Figure 3B). Next, we calculated the fractions of interactions involving each residue for all four types of interactions with the ligand. Results demonstrate a stable binding mode primarily driven by a robust hydrogen bond network involving residues Asp231, Gln228, and Val292, further stabilized by water-mediated contacts with Glu177 and Asp156 (Figure 3C–E). Collectively, these in silico findings highlight Jionoside B1 as a promising lead compound and warrant further experimental validation.

### 3.4. Jionoside B1 Binds SIRT3 with High Affinity and Inhibits Its Enzymatic Activity

**Figure 4:**
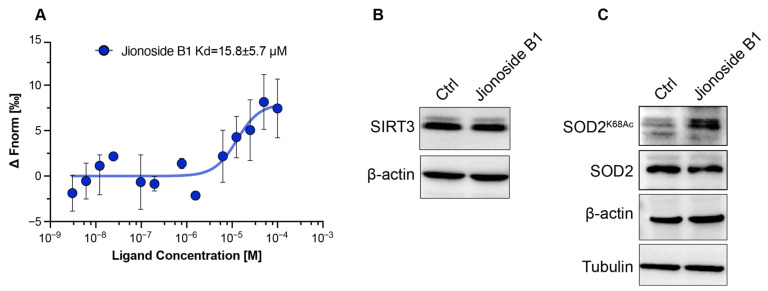
Jionoside B1 Binds SIRT3 with High Affinity. (**A**) MST analysis of the binding affinity of SirT3 with Jionoside B1. (**B**) Western blot analysis of SIRT3 protein expression in MDA-MB-231 cells treated with Jionoside B1 (10 µM) for 12 h. (**C**) Western blot analysis of lysines K68 acetylation in MDA-MB-231 cells treated with Jionoside B1 (10 µM) for 12 h.

To validate the binding activity of candidate compounds with SIRT3, we employed microscale thermophoresis (MST) to determine the dissociation constants (Kd) between the candidate compounds and SIRT3. MST results revealed that Jionoside B1 displayed a Kd value of 15.8 ± 5.7 μM, indicating that Jionoside B1 had a high binding affinity with SIRT3 (Figure 4A). SIRT3 directly deacetylates the K68 site of SOD2 in an NAD^+^-dependent manner within mitochondria; therefore, the acetylation level of SOD2 K68 serves as a direct indicator of SIRT3 enzymatic activity. Western blot analysis demonstrated that Jionoside B1 did not alter SIRT3 protein expression levels but significantly inhibited SIRT3 enzymatic activity, thereby enhancing the acetylation level of SOD2 K68 (Figure 4B,C). Collectively, these results indicate that Jionoside B1 effectively binds to SIRT3 and effectively inhibits its enzymatic activity.

### 3.5. Jionoside B1 Enhances Cisplatin Sensitivity In Vitro

**Figure 5:**
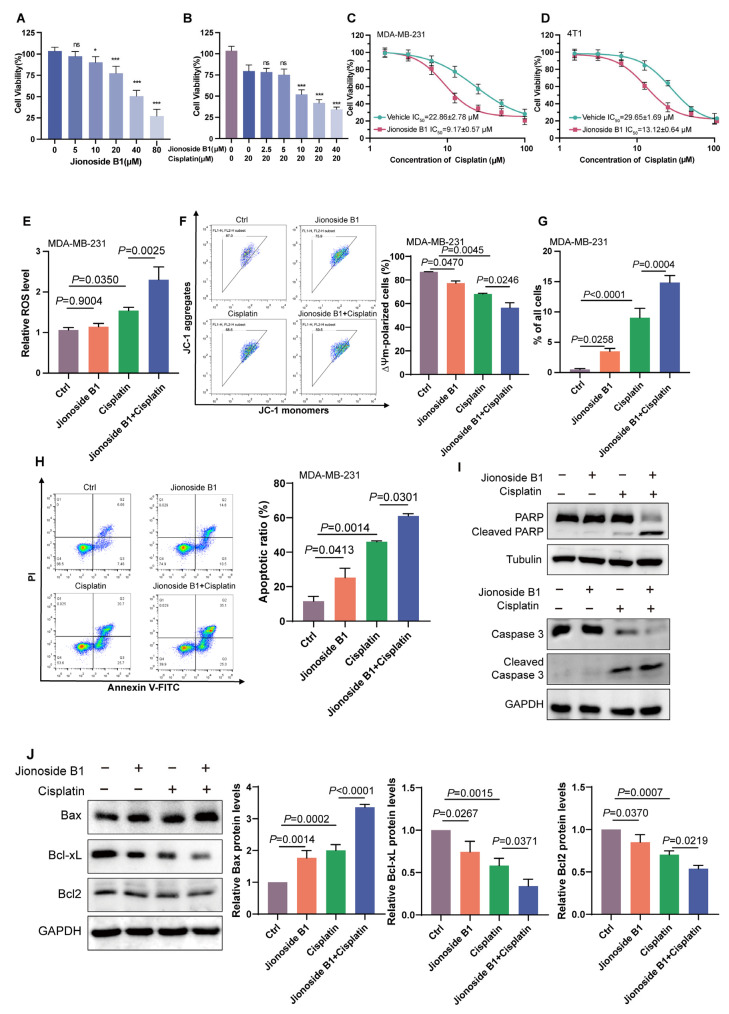
Jionoside B1 Enhances Cisplatin Sensitivity in vitro. (**A**) Cell viability after treatment with increasing concentrations of Jionoside B1 (0–80 µM). Data are presented as mean ± SEM. ns, not significant; * *p* < 0.05; *** *p* < 0.001. (**B**) Cell viability after treatment with cisplatin (20 µM) and different concentrations of Jionoside B1 (0–40 µM). Data are presented as mean ± SEM. ns, not significant; *** *p* < 0.001. (**C**) Dose–response curves of cisplatin (1–100 µM) in MDA-MB-231 cells treated with vehicle or Jionoside B1. (**D**) Dose–response curves of cisplatin (1–100 µM) in 4T1 cells treated with vehicle or Jionoside B1. (**E**) Relative intracellular ROS levels in MDA-MB-231 cells treated with Jionoside B1 (10 µM), cisplatin (20 µM), or combination. (**F**) Representative JC-1 flow-cytometry plots and quantification of ΔΨm-polarized cells under the indicated treatments. (**G**) Percentage of sub-G1 cells in MDA-MB-231 cells treated with Jionoside B1 (10 µM), cisplatin (20 µM), or combination for 12 h using PI staining. (**H**) Flow cytometry plots showing apoptosis analysis of MDA-MB-231 cells treated with Jionoside B1 (10 µM), cisplatin (20 µM), or combination for 24 h using Annexin V-FITC/PI staining. (**I**) Western blot analysis of PARP, cleaved PARP, caspase-3 and cleaved caspase-3 in MDA-MB-231 cells treated with Jionoside B1 (10 µM), cisplatin (20 µM), or combination for 12 h. (**J**) Western blot analysis and quantification of Bax, Bcl-xL and Bcl2 protein expression in MDA-MB-231 cells treated with Jionoside B1 (10 µM), cisplatin (20 µM), or combination for 12 h.

Previous studies have shown that SIRT3 maintains mitochondrial integrity and functional homeostasis by deacetylating mitochondrial proteins, thereby regulating the mitochondria-dependent apoptotic pathway. Cohort studies have also confirmed a strong correlation between SIRT3 expression and chemotherapeutic efficacy. Therefore, the SIRT3-specific inhibitor we identified may enhance tumor cell sensitivity to chemotherapeutic agents. Initially, we evaluated the cytotoxicity of Jionoside B1 alone and found that it inhibited cell viability in a dose-dependent manner (Figure 5A). Subsequently, we investigated the combination strategy by treating cells with a fixed concentration of cisplatin (20 μM) alongside increasing doses of Jionoside B1. While lower doses (2.5 and 5 μM) showed no significant additive effect, Jionoside B1 concentrations ranging from 10 μM to 40 μM significantly enhanced cisplatin-induced cytotoxicity (Figure 5B). Considering that 10 μM Jionoside B1 exhibited only mild toxicity on its own yet significantly enhanced cisplatin efficacy, this concentration was selected for subsequent sensitization studies. Consistently, the addition of 10 μM Jionoside B1 markedly increased the sensitivity of MDA-MB-231 and 4T1 cells to cisplatin, reducing IC50 values from 22.86 ± 2.78 μM and 29.65 ± 1.69 μM to 9.17 ± 0.57 μM and 13.12 ± 0.64 μM, respectively (Figure 5C,D). Further investigation demonstrated that Jionoside B1 treatment significantly elevated intracellular ROS levels and decreased mitochondrial membrane potential (Figure 5E,F). To further validate the pro-apoptotic effects, we performed cell cycle analysis and flow cytometry-based apoptosis assays. Cell cycle profiling revealed that the combination treatment markedly increased the proportion of Sub-G1 phase cells from approximately 9% in the cisplatin-alone group to 15% in the Jionoside B1 plus cisplatin group in MDA-MB-231 cells (Figure 5G and Figure S2A). Consistent with this observation, flow cytometry analysis using Annexin V/PI staining demonstrated that the proportion of apoptotic cells increased from 42% in the cisplatin-alone group to 61% in the combination treatment group (Figure 5H and Figure S2B). Western blot analysis showed that the combination of Jionoside B1 and cisplatin significantly decreased Bcl2 expression, increased Bax and Cytochrome c expression, and enhanced caspase3 and PARP cleavage (Figure 5I,J and Figure S2C). Together, these results indicate that Jionoside B1 potentiates chemotherapy-induced mitochondrial apoptosis by inhibiting SIRT3 activity, thereby enhancing tumor cell sensitivity to chemotherapeutic agents.

### 3.6. Jionoside B1 Enhances Cisplatin Sensitivity In Vivo

**Figure 6:**
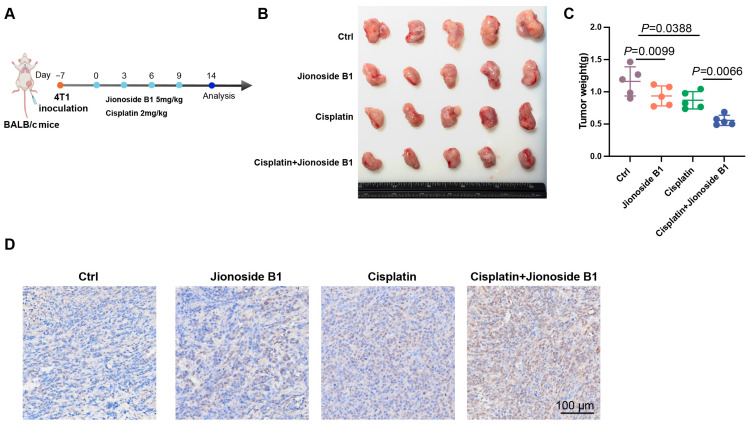

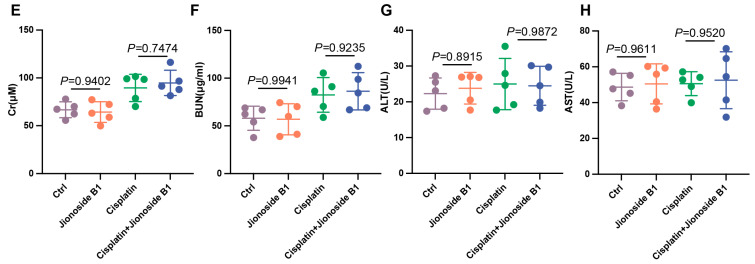
Jionoside B1 Enhances Cisplatin Sensitivity in vivo. (**A**) Scheme and timeline showing the experimental design to evaluate the effect of different treatments in a 4T1 tumor model. (**B**) Representative photographs of excised tumors from four treatment groups. (**C**) Terminal tumor weights in mice after different treatments (*n* = 5). (**D**) Representative immunohistochemical analysis of cleaved PARP expression in tumor sections. Scale bar, 100 μm. (**E**,**F**) Serum creatinine (Cr; (**E**)) and blood urea nitrogen (BUN; (**F**)) levels were measured to assess renal function in mice after the indicated treatments (*n* = 5). (**G**,**H**) Serum alanine aminotransferase (ALT; (**G**)) and aspartate aminotransferase (AST; (**H**)) levels were measured to assess liver function in mice after the indicated treatments (*n* = 5).

In vitro experiments have demonstrated that Jionoside B1 markedly enhances tumor sensitivity to cisplatin. To further evaluate its pharmacological activity in vivo, an orthotopic breast cancer model was established by orthotopic implantation of 4T1 murine mammary carcinoma cells (Figure 6A). Intraperitoneal administration of Jionoside B1 (5 mg/kg) significantly increased the antitumor efficacy of cisplatin. Compared with the cisplatin monotherapy group, the combination group exhibited significantly reduced tumor weight (Figure 6B,C). Immunohistochemical analysis of tumor tissues further revealed a marked increase in cleaved PARP expression in the combination group (Figure 6D). Additionally, no significant differences in renal function markers (Cr, BUN) or liver enzymes (ALT, AST; G-H) were observed between the Jionoside B1-treated group and the control group. Furthermore, combining Jionoside B1 with cisplatin did not significantly alter these safety markers compared to cisplatin alone, indicating that Jionoside B1 exhibits a favorable safety profile (Figure 6E–H). Overall, these findings indicate that Jionoside B1 significantly enhances cisplatin-induced apoptosis in vivo, thereby increasing tumor sensitivity to chemotherapy.

## 4. Discussion

In this study, we found that SIRT3 was highly expressed in breast cancer patients, and patients with high SIRT3 expression exhibited shorter overall survival and reduced sensitivity to chemotherapeutic agents compared to those with low SIRT3 expression, suggesting that SIRT3 represents a potential therapeutic target in breast cancer. To this end, we utilized molecular docking-based virtual screening to identify Jionoside B1 from a natural product library as a potential SIRT3 inhibitor. Microscale thermophoresis (MST) assays verified the binding affinity of this compound to SIRT3, with a Kd value of 15.8 ± 5.7 μM. Western blot analysis further confirmed that Jionoside B1 significantly inhibited SIRT3 activity, leading to increased acetylation of SOD2 at Lys68. In vitro experiments demonstrated that Jionoside B1 enhanced the sensitivity of breast cancer cell lines MDA-MB-231 and 4T1 to cisplatin by suppressing SIRT3-mediated ROS scavenging, disrupting mitochondrial functional homeostasis, and ultimately inducing apoptosis. Consistent with these findings, in vivo experiments revealed that Jionoside B1 increased the antitumor efficacy of cisplatin, with combined administration significantly inhibiting tumor growth in a 4T1 orthotopic breast cancer model.

Recent studies indicate that natural products can serve as effective adjuvants to cisplatin by exerting direct anticancer activity and enhancing cisplatin efficacy through multi-target mechanisms. Compounds such as curcumin and quercetin suppress NF-κB signaling, reduce anti-apoptotic proteins (e.g., Bcl-2 and survivin), and thereby promote apoptosis [40]. Isorhamnetin and azadirachtin attenuate Akt phosphorylation, helping to overcome cisplatin resistance and increase cisplatin-induced cell death. In addition, curcumin and other plant extracts can inhibit xCT/SLC7A11, deplete intracellular glutathione (GSH), and trigger ferroptosis [41]. Moreover, natural compounds including quercetin and glycyrrhizin may downregulate P-glycoprotein, limit drug efflux and increase intracellular cisplatin accumulation [42]. In this context, our identification of Jionoside B1 as a SIRT3 inhibitor represents a novel mechanism by which natural products can synergize with cisplatin to enhance therapeutic efficacy in breast cancer.

Despite these promising findings, our study has several limitations that should be acknowledged. First, while MST confirmed the physical binding of Jionoside B1 to SIRT3 and Western blot analysis showed increased acetylation of the canonical substrate SOD2 (K68), we did not perform a direct cell-free enzymatic assay. Thus, SIRT3 inhibition is inferred from substrate acetylation status rather than direct catalytic kinetics. Future studies utilizing direct deacetylase activity assays will be valuable to further corroborate the enzymatic inhibition mechanism. Moreover, although molecular docking and functional assays point to SIRT3 as a primary target, natural products can sometimes exhibit off-target effects. While our rescue experiments support SIRT3 specificity, comprehensive proteomic profiling would be beneficial to fully exclude interactions with other mitochondrial proteins.

Second, although our results support oxidative stress-associated mitochondrial dysfunction and caspase-dependent apoptosis as the predominant mechanism in this system, SIRT3 also regulates other stress-adaptive programs, including ferroptosis and pro-survival signaling (e.g., PI3K/Akt/mTOR) [19,20]. Because we did not directly assess ferroptosis (e.g., lipid peroxidation and the GPX4 axis) or measure Akt/mTOR pathway activation, we cannot exclude contributions from these processes to the response to Jionoside B1 plus cisplatin. Future studies evaluating ferroptotic markers and PI3K/Akt/mTOR pathway activity will help determine whether these processes are also involved in the chemosensitization observed in our model.

Third, the in vivo doses of Jionoside B1 and cisplatin used in this study were suitable for testing whether pharmacologic SIRT3 inhibition enhances cisplatin activity; however, synergy-guided dose optimization and dose-reduction strategies to better substantiate synergism and mitigate toxicity will require systematic dose-matrix studies and should be performed in future investigations.

Finally, we observed that Jionoside B1 significantly enhances cisplatin sensitivity in MDA-MB-231 and 4T1 cells, effectively reducing the required cytotoxic dose. Given that reduced drug sensitivity often represents an early step toward the development of acquired resistance, these findings raise the possibility that Jionoside B1 may also modulate resistance-related phenotypes. Although we did not test cisplatin-resistant breast cancer cell lines, the ability of Jionoside B1 to increase cisplatin sensitivity in parental models suggests that it may also have the potential to counteract resistance-associated phenotypes. Future studies using paired cisplatin-sensitive and -resistant models will be required to directly test whether Jionoside B1 can reverse established cisplatin resistance.

In conclusion, this study not only elucidates the critical role of SIRT3 in modulating chemotherapy sensitivity in TNBC but also successfully identifies Jionoside B1 as a novel and potent SIRT3 inhibitor. These findings provide a promising combination therapeutic strategy to enhance cisplatin sensitivity in TNBC. Our results validate SIRT3 as a viable therapeutic target and support the potential of combining SIRT3 inhibition with cisplatin to improve cisplatin responsiveness, highlighting potential for clinical translation.

## Figures and Tables

**Table 1:** The virtual screening hit information towards SIRT3.

Rank	Title	Docking Score
1	Jionoside B1	−12.777
2	Echinacoside	−12.697
3	Magnoloside F	−11.73
4	Cassiaside B	−11.7
5	Forsythoside F	−11.696
6	Cassiaside B2	−11.65
7	Forsythoside B	−11.625
8	Dactylorhin A	−11.606
9	1,2,3,6-Tetragalloylglucose	−11.511
10	Parishin B	−11.482
11	Jionoside A1	−11.447
12	Cistanoside A	−11.427
13	Rubrofusarin gentiobioside	−11.37
14	Nor-rubrofusarin gentiobioside	−11.369
15	Parishin C	−11.323
16	Magnoloside B	−11.275
17	Tubuloside A	−11.269
18	Levomefolic Acid	−11.238
19	Poliumoside	−11.205
20	Benzoylpaeoniflorin	−11.007
21	Pentagalloylglucose	−10.846
22	Camelliaside A	−10.838
23	Myricoside	−10.833
24	11-oxo-mogroside V	−10.825
25	Clerodendrin	−10.802
26	Verbascoside	−10.674
27	Secoisolariciresinol diglucoside	−10.661
28	Angoroside C	−10.653
29	Neohesperidin Dihydrochalcone	−10.644
30	Naringin dihydrochalcone	−10.569
31	Dauricine	−10.488
32	Isoliquiritin apioside	−10.427
33	Plantagoside	−10.404
34	Myricitrin	−10.397
35	Brandioside	−10.356
36	Terrestrosin D	−10.353
37	Complanatoside B	−10.341
38	Clitorin	−10.291
39	Plantamajoside	−10.238
40	Militarine	−10.233

## Data Availability

Data is contained within the article or Supplementary Material.

## Supplementary Materials

The following supporting information can be downloaded at: https://www.mdpi.com/article/10.3390/biomedicines14020421/s1, Figure S1: Supplemental data for Figure 1: SIRT3 is overexpressed in BRCA and is associated with poor overall survival and chemotherapy sensitivity. (A) Expression of SIRT3 in different subtype of BRCA samples from METABRIC cohort. (B) The correlation between SIRT3 expression and overall survival in different subtype of BRCA samples from METABRIC cohort.; Figure S2: Supplemental data for Figure 5: Jionoside B1 Enhances Cisplatin Sensitivity in vitro. (A) Flow cytometry plots showing cell cycle analysis of MDA-MB-231 cells treated with Jionoside B1, cisplatin, or combination for 12 hours using PI staining. (B) MDA-MB-231Cells were analyzed by flow cytometry without Annexin V-FITC/PI staining to establish gating parameters and baseline fluorescence. (C) Western blot analysis of Cytochrome C in MDA-MB-231 cells treated with Jionoside B1(10 µM), cisplatin (20 µM), or combination for 12 hours.
