# Supplementary material for: Jionoside B1 Sensitizes TNBC to Cisplatin by Inhibiting SIRT3-Mediated Oxidative Stress Defense
Source: Biomedicines. 2026 Feb 13;14(2):421. doi: 10.3390/biomedicines14020421 (PMC12938267; doi:10.3390/biomedicines14020421)
Supplement: Supplementary file 1 [file biomedicines-14-00421-s001.zip › biomedicines-4116098-supplementary.pdf]

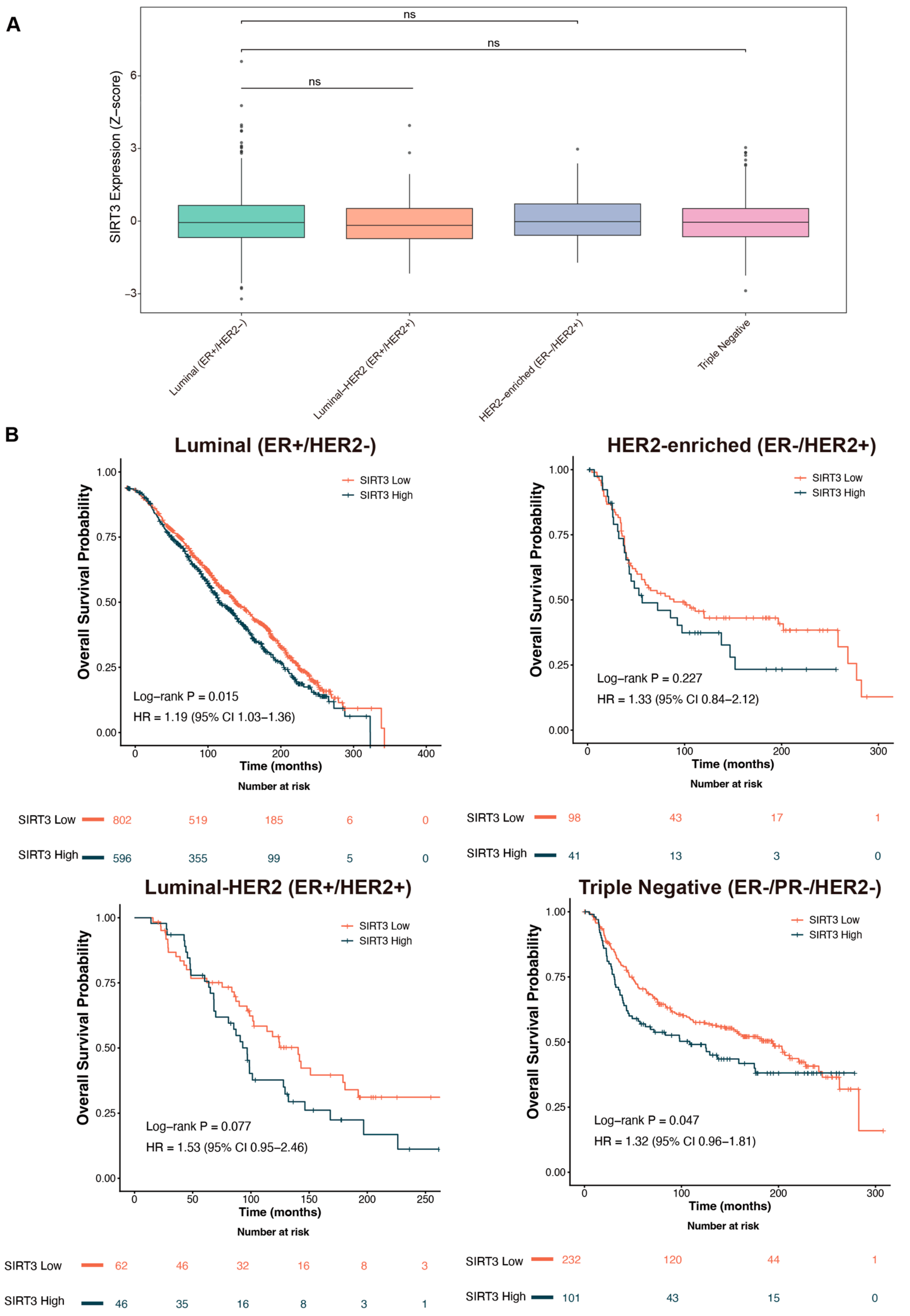

Figure.S1 Supplemental data for Figure 1: SIRT3 is overexpressed in BRCA and is associated with poor overall survival and chemotherapy sensitivity. (A) Expression of SIRT3 in different subtype of BRCA samples from METABRIC cohort. (B) The correlation between SIRT3 expression and overall survival in different subtype of BRCA samples from METABRIC cohort.

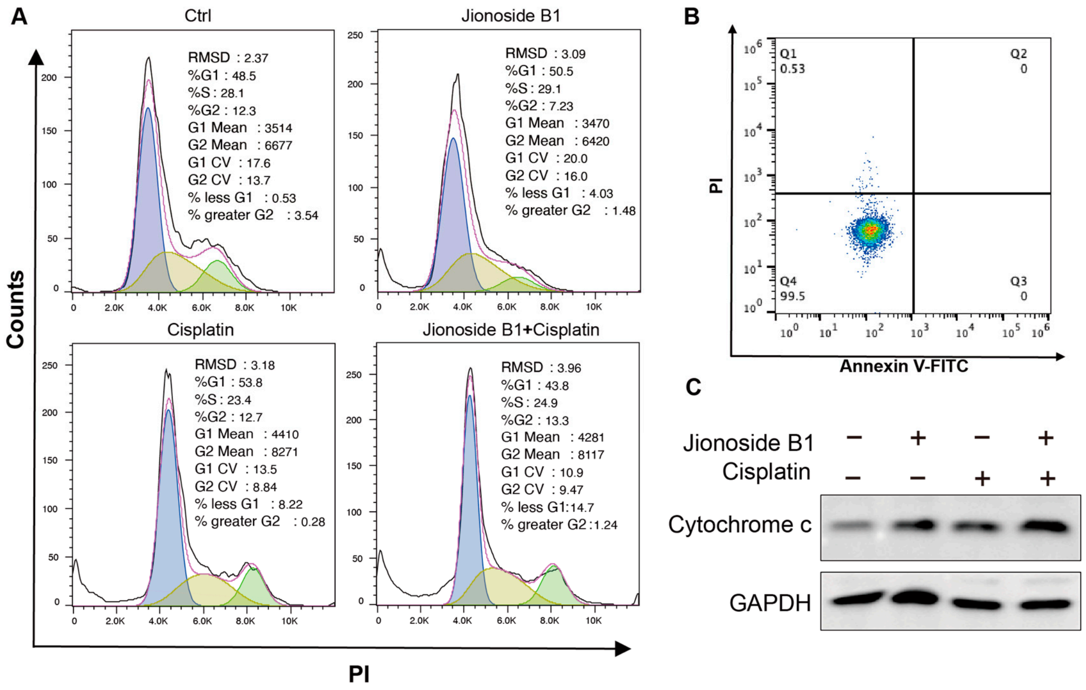

Figure.S2 Supplemental data for Figure 5: Jionoside B1 Enhances Cisplatin Sensitivity in vitro. (A) Flow cytometry plots showing cell cycle analysis of MDA-MB-231 cells treated with Jionoside B1, cisplatin, or combination for 12 hours using PI staining. (B) MDA-MB-231Cells were analyzed by flow cytometry without Annexin V-FITC/PI staining to establish gating parameters and baseline fluorescence. (C) Western blot analysis of Cytochrome C in MDA-MB-231 cells treated with Jionoside B1(10 μM), cisplatin (20 μM), or combination for 12 hours.
